# Foraging strategy of a carnivorous-insectivorous raptor species based on prey size, capturability and nutritional components

**DOI:** 10.1038/s41598-020-64504-4

**Published:** 2020-05-05

**Authors:** Juan A. Fargallo, Juan Navarro-López, Patricia Palma-Granados, Rosa M. Nieto

**Affiliations:** 1 Departamento de Ecología Evolutiva, Museo Nacional de Ciencias Naturales-CSIC, José Gutiérrez Abascal 2, 28006 Madrid, España; 2 Department of Physiology and Biochemistry of Animal Nutrition, Estación Experimental del Zaidín-CSIC, Camino del Jueves s/n, 18100 Armilla Granada, España

**Keywords:** Ecology, Evolutionary ecology, Zoology, Animal behaviour

## Abstract

Optimal foraging theory has typically paid little attention to species feeding on mobile prey and has emphasised energy intake rather than the nutritional contribution of food. The difficulty of capturing food has rarely been included in foraging models, even when it is a potentially important modulator of time devoted to foraging. From the central place foraging and provisioning perspectives, it is posited that at high levels of prey selectivity, the time spent to capture prey is longer than at low levels of prey selectivity. Furthermore, in the case of carnivorous predators, it is thought that nutritional composition does not influence foraging strategies. To explore these issues, we investigated the influence of abundance, size, difficulty of capture, gross energy and nutritional composition (fat, protein, protein-fat ratio and amino acid contents) of prey species on the foraging behaviour of a predator species, the common kestrel *Falco tinnunculus*, in a region of high diversity of prey species. Our results show that capturability index and load-size explain the foraging behaviour of kestrels. Preferred prey take longer to be provisioned, both selectivity and capturability might explain this result. It is also shown that specific nutritional components, such as protein and amino acid contents, are likely to explain food preference in this carnivorous-insectivorous species.

## Introduction

Optimal foraging theory focusses on determining the efficiency of individuals in obtaining food resources required for survival and reproduction^1^. Foraging is one of the most commonly modelled behaviours in ecology research, with current models including a wide array of variables ranging from predator and prey behaviour to prey abundance and population dynamics^2^. Nonetheless, energy (expenditure and intake) and the time spent on foraging activity have remained the pillars of the optimal foraging theory since its inception^3,4^. More effort is needed to obtain empirical data in the wild, as some theoretical assumptions have been revealed to be erroneous when tested in the field. This has led to criticisms^5^ and even harsh disagreements regarding the heuristic capacity of the theory^6^. For example, the theory has failed when applied to species feeding on mobile prey, such as predators, or when placing excessive emphasis on energy intake instead of nutritional composition^7,8^.

Parent birds provisioning their offspring at the nest have been a typical study model to explore foraging strategies usually under the “central place” and “provisioning” schemes. This provisioning behaviour represents the common case of foragers that return to a fixed location between foraging trips to feed young, store food, or rest, and are thus hindered by spatial constraints on foraging^9–12^. Like other behavioural models, these provisioning models emphasise the ways in which costs and benefits select for certain types of behaviour. Optimal foraging is achieved by minimising travel distance and time through selecting the foraging patches nearest to the nest, on the one hand, and selecting the most energy-efficient food on the other. In other words, the food with the greatest energy content per hunting time is selected. Foraging time will depend on the individual efficiency in searching, accessing/capturing and handling food, which in turn depends on the individual’s inherited/learned foraging behaviour (reviewed by^12^). It is also thought that prey selectivity affects the between-feeding interval and therefore feeding rate. A more restrictive selection requires more time devoted to finding and capturing preferred prey, which increases the time devoted to provisioning the nest with food, with preferred food requiring longer between-feeding intervals^13^. In the case of predators, foraging is the result of a co-evolutionary arms race between the hunting strategies of the predators and the antipredatory behaviour of the prey species^14–16^. The foraging efficiency of predators depends on morphological and behavioural adaptations to capturing prey, but also on the ecological characteristics and morphological traits (including size) of prey species derived from antipredatory strategies^17–19^, for which the observed preference for food is linked to the efficiency in obtaining it^20,21^. Ecological characteristics vary among localities as do ecological conditions. However, although seemingly relevant, many studies ignore the variation in the difficulty of capturing different prey species based on their ecological characteristics, which may affect searching and handling time and prey selectivity or preference, and therefore, feeding rate^22^. This is especially relevant for species or populations with a broad trophic niche.

Regarding the foraging strategies of carnivorous predators, the traditional view has assumed that searching for nutritional components is unnecessary in this group^8^. While for herbivores and omnivores it is thought that food differs in its nutritional composition, in the case of carnivores it is assumed that nutrient content is similar for different prey species guaranteeing nutrients relative to their requirements^23,24^ (reviewed by^8^). However, whereas the energy in prey has long been hypothesised as the main prey feature modulating the foraging behaviour of predators^25^, it has now been shown that nutrients in the diet also influence their foraging decisions^26–30^, (but see^31^).

A large amount of prey biomass (large size or high quantity) does not guarantee the presence of essential nutrients needed for optimal growth or self-maintenance^32^. Deficits in nutritional elements of the diet, such as of some amino acids, lipids or proteins, have been observed to reduce body condition, growth rates, reproduction and survival probability^33–36^. Recently, it has been reported that individuals are able to regulate their intake of multiple nutrients independently by choosing specific prey types and diets^8,33,37–40^. A well-adjusted protein and fat composition in food may improve the expression of life-history traits, such as immune function^41,42^, sexual display^43^, body size and growth rate^33,39,44^, suggesting strong selection for a given nutrient composition in the diet. Furthermore, an equilibrium between proteins and lipids (i.e., the proportional contribution of protein to energy (protein-fat ratio)), has long been considered a key nutritional element for animal growth^45,46^. In addition, amino acids (AA) are not simply protein precursors, but also participate in and regulate key metabolic pathways necessary for growth, reproduction and immunity^47^. These so-called functional AA include some essential amino acids (EAA), sulphur-containing AA (SAA) and branched-chain AA (BCAA)^47,48^. EAA cannot be synthesised de novo by animals and, therefore, must be acquired through the diet. An AA disproportion in protein nutrition affects health and provokes reduced food intake, abnormal behaviour and impaired growth in animals^47^. SAA (cysteine and methionine) are involved in the metabolism of proteins, development of the immune system, synthesis of melanin pigments and synthesis of glutathione and taurine, which are essential for defence against oxidative stress^49–51^. BCAA (leucine, isoleucine and valine) have been described to enhance glucose consumption, increase protein synthesis in skeletal muscle, prevent oxidative damage, regulate lipolysis and upregulate immune responses^52,53^.

The balanced-diet hypothesis^54,55^ posits that broadening trophic niche supplies a more complete range of nutrients, providing fitness benefits to the consumer^55,56^. Adaptive strategies for optimal or maximum growth should involve selectiveness of particular nutrients over others to provision offspring, although this proximately depends on the difficulty of obtaining food of contrasting nutritional value and on the capacity of parents to procure it from their territories^57–59^.

Raptors are a taxonomic group in which feeding has been intensively studied. Nevertheless, it is striking how little their foraging behaviour has been analysed within the framework of provisioning, or the role of prey capturability and nutritional demands in their foraging strategies.

In this study, we examined food provisioning to the nest in common kestrels *Falco tinnunculus*, a small raptor species that has been traditionally considered a vole specialist in northern latitudes^60,61^, while its diet is made up of a wide range of vertebrate and invertebrate prey species in more southern latitudes^62^. We measured the average time that kestrels take to bring each prey species (mean between-feeding interval) using prey provisioning recordings made over nine breeding seasons. Moreover, we estimated abundance, preference, capturability, energy (calories) and nutritional composition (fat, protein and amino acid contents) of the prey species consumed by kestrels. In a first step, we explored the foraging behaviour of kestrels when provisioning the nest with food by analysing how prey size (weight) and the difficulty of capturing the prey influence the time that kestrels take to capture and bring a prey to the nest. In addition, in the context of provisioning, we tested the hypothesis that more selective foraging on preferred prey species will result in longer provisioning times^13^ by analysing the relationship between the time taken to provision a prey and prey preference. Finally, regarding the nutritional perspective, we analysed whether kestrel preference is influenced by the nutritional composition of the prey (protein, fat, amino acid contents, gross energy and protein-fat ratio).

## Methods

### Study area

Field work was carried out in the region of Campo Azálvaro in central Spain. The study area is a treeless flat valley at 1,300 m above sea level, mainly devoted to cattle-raising (see^62^ for habitat description). About 20–45 breeding pairs of wild kestrels nest each year in 62 artificial nest boxes installed in the study area^63^.

### Prey provisioning time

During the kestrel breeding seasons of 2006–2014, the prey items provided by parents to their chicks were recorded in 202 nests. Recordings were made at 12–14 days of age of the chicks by placing a digital camera in the nest box^62^. Recording was continuous for 24 hours or more without researcher interruptions, although some nests were not filmed for the entire period due to technical problems^62^. Recordings were displayed in the free VLC Media Player software (www.videolan.org) to identify each delivered prey item. Between-feeding intervals were also noted to estimate the “prey provisioning time” (PPT), defined as the mean time elapsed from the previous delivered prey item of any species to the next for a given prey species; that is, the mean feeding interval for the target prey species and controlling for time of day, between-year and between-nest variation (see below). Feeding intervals were recorded for a total 12,779 prey items.

#### Prey weight

The body mass of prey species was calculated as the mean body mass of individuals captured in our study area or the mean body mass described for the species in the literature when no data in our study area could be collected (see^53^ for more details). In the case of the ocellated lizards *Timon lepidus*, prey remains found in the nests are not representative of the maximum sizes found in the lizard population of the study area (unpublished data), for which reason the weight was estimated on the basis of the cranium length of the individuals found in kestrel nests as prey remains (n = 113). Cranium length explained 90% of the variance in body mass of the ocellated lizards that we trapped in the study area (r = 0.95, F_1,64_ = 590.68, P < 0.001, n = 66). The mean weight of lizards consumed by kestrels was then calculated using the function: *weight* = *−79.55* + 4.81 * cranium length, extracted from the linear regression.

### Prey capturability index

Potential capturability of prey species was calculated by assigning values to those ecological features that impede (or facilitate) prey capture by kestrels. We used eight ecological features: three refer to the environment in which the prey species live, two to prey availability and three to the behavioural antipredatory response. Three ecological characteristics (habitat protection, location and abundance) were based on observations made in our particular study area. For all features, values varied between 1 and 3, with 3 expressing the highest level of difficulty of capture. Prey capturability was therefore defined as the sum of the eight values, with higher values indicating a higher difficulty of capture (Table 1). The ecological characteristics were classified as follows:

**Table 1:** Values assigned to each ecological feature of the prey. A = medium of displacement, B = surface-subterranean way of life, C = habitat protection in foraging areas, D = abundance, E = location, F = type of escape, G = speed flight and H = refuge use.

Prey species	A	B	C	D	E	F	G	H	Capturability index
*Microtus arvalis*	1	2	2	3	2	1	2	2	15
*Crocidura russula*	1	2	2	3	2	1	2	2	15
*Alauda arvensis*	3	1	1	3	1	3	3	1	16
*Sturnus unicolor*	3	1	1	3	2	3	3	1	17
*Chalcides striatus*	1	1	2	2	2	1	3	2	14
*Timon lepidus*	1	1	1	3	2	1	3	2	14
*Lacerta schreiberi*	1	1	2	3	3	1	3	2	16
*Psammodromus hispanicus*	1	1	1	2	1	1	2	2	11
*Pelophylax perezi*	2	1	2	3	2	2	2	3	17
*Lycosa tarantula*	1	3	3	1	1	1	1	3	14
Caterpillar	1	1	1	1	1	1	1	1	8
*Gryllus campestris*	1	2	1	1	1	1	1	3	11
*Gryllotalpa gryllotalpa*	1	3	3	1	1	1	1	3	14
*Acrididae-Tettigoniidae*	2	1	1	1	1	2	2	1	11
Coleoptera*	2	1	1	1	1	1	1	1	9

(*) Values given to the coleopteran Order are based on the main families and species observed as prey in kestrel nests and recordings: *Scarabeidae* (*Scarabeinae*, *Cetoniinae* and *Melolonthinae*) and *Carabidae*.

A) Medium of displacement (1 = terrestrial, 2 = terrestrial-aquatic or terrestrial-aerial and 3 = mainly aerial).

B) Surface-underground way of life (1 = epigean, 2 = fossorial and 3 = subterranean).

C) Habitat protection in foraging areas (1 = exposed areas with short vegetation (mainly pastured lands), 2 = areas covered by vegetation and 3 = underground).

D) Abundance (1 = very abundant, 2 = abundant and 3 = not abundant). See Supplementary Appendix S1.

E) Location with respect to the kestrel breeding area (1 = present throughout breeding area and surroundings, 2 = located within the breeding area and 3 = located outside of the breeding area). Breeding area was defined as the area where kestrel nest boxes were installed (23 km^2^), considering a radius of 1000 m around the nest box^62^.

F) Type of escape (1 = walking-running, 2 = jumping, 3 = flying).

G) Speed flight (1 = slow, 2 = fast and 3 = very fast).

H) Use of refuges (1 = not used, 2 = distant to the refuge and 3 = close to the refuge).

### Nutritional composition of prey species

Nutrient (crude protein, lipids, ash and amino acids) and energy contents were assessed in the 11 main prey species (Table 2) or prey groups. These 11 prey species-groups make up 91% of the kestrel diet^62^ (Supplementary Table S1). In addition, the nutritional composition of kestrel diet ate the population level was determined by considering the mean nutritional composition of consumed prey species and the total biomass (grams) provided by each (weighted arithmetic mean; Table 2).

**Table 2:** Weight and analyses of nutrient composition of prey species of kestrels. Water, fat and protein (nitrogen × 6.25) are expressed as g/100 g fresh prey weight (wet mass). Energy content is given as kJ per g fresh weight. P:F is the protein-fat ratio. Also mean values for kestrel diet are shown.

Prey species	Weight (g)	Water (%)	Protein (%)	Fat (%)	P:F	KJ/g
*Microtus arvalis*	31.3	76.4	18.2	1.7	10.5	5.1
*Crocidura russula*	8.1	71.9	21.4	3.3	6.5	6.3
Birds *(Alauda arvensis* + *Sturnus unicolor)*	51.8*	69.0	22.1	3.7	6.0	7.0
*Chalcides striatus*	13.3	70.3	18.2	4.6	4.0	6.1
*Timon lepidus*	52.2**	74.9	19.8	2.1	9.3	5.3
*Lacerta schreiberi*	29.4	75.4	20.2	1.2	16.6	5.1
*Psammodromus hispanicus*	2.7	74.9	19.0	2.5	7.7	5.5
*Pelophylax perezi*	32.7	78.7	16.7	0.8	21.1	4.3
*Gryllus campestris*	1.2	77.9	16.2	1.6	10.4	5.1
*Gryllotalpa gryllotalpa*	2.4	69.6	20.2	3.2	6.3	6.8
*Acrididae-Tettigoniidae*	1.2	74.4	19.1	2.1	9.3	6.0
**Kestrel diet**	**7.2**	**74.4**	**19.1**	**2.3**	**8.3**	**5.6**

(*) Weight of Eurasian skylarks *Alauda arvensis* + spotless starlings *Sturnus unicolor* were calculated through the weighted arithmetic mean considering the occurrence of each species in the kestrel diet.

(**) Ocellated lizard *Timon lepidus* weight was estimated using the size of the lizards found in kestrel nests as prey remains.

Nutritional values for vertebrates (birds, reptiles and mammals) come from freshly hunted prey found in kestrel nests at the time they were visited. Removed prey items were replaced by commercial dead rooster chicks to avoid food deprivation. In the case of birds, nutritional values of spotless starlings *Sturnus unicolor* and Eurasian skylarks *Alauda arvensis* were analysed together. These two species represent 77% of the bird species consumed by kestrels^62^. Invertebrates (field crickets, mole crickets, bush crickets and grasshoppers) are rarely found in kestrel nests because of their small size, for which reason permissions were granted by the environmental office of the Junta de Castilla y León to collect specimens in the field. Nutritional values for the *Acrididae-Tettigoniidae* group come from several different species of both Orthoptera families. Collected prey items were stored at −20 °C until freeze-drying and grinding with liquid nitrogen (Retsch Ultra Centrifugal Mill ZM 200; Restch GmbH, Haan, Germany) before analysis for nutritional composition. Because kestrels remove the large feathers of birds (remiges and rectrices) prior to consumption, the same was done for the analyses. Approximately 10 grams of freeze-dried material were used in each prey species. Several specimens of the same species were homogenised together for this purpose. In total, 14 common voles *Microtus arvalis*, 9 common shrews *Crocidura russula*, 2 Eurasian skylarks (fledglings), 2 spotless starlings (fledglings), 6 three-toed skinks *Chalcides striatus*, 6 ocellated lizards *Timon lepidus*, 6 Schreiber’s lizards *Lacerta schreiberi*, 26 psammodromus lizards *Psammodromus hispanicus*, 3 Perez’s frogs *Pelophylax perezi*, 54 field crickets *Gryllus campestris*, 14 mole crickets *Gryllotalpa gryllotalpa* and 56 *Acrididae-Tettigoniidae* were used. All samples were collected in 2014.

All analyses were performed in duplicate. The analytical methods are described by^64^. Briefly, dry matter and total ash content were analysed according to the AOAC^65^ (method 934.01 and method 942.05). Dry matter was determined using an aliquot sample of freeze-dried material to establish the residual water content after freeze-drying. Total N was determined by combustion, according to Dumas’ method, in a TruSpec CN analyser (Leco Corporation, St. Joseph, MI). Crude protein content was calculated using a factor of 6.25. Fat content was determined by ether extraction according to standard procedures^65^. The energy content was determined in an isoperibolic bomb calorimeter (Parr Instrument Co., Moline, IL) and expressed as kilojoules (kJ) per gram of each prey species as described by^64^. Briefly, the increase in water temperature is determined in a container, in which a known amount of sample −0.5 to 1.0 g (± 0.1 mg)- is combusted completely in a high oxygen concentration atmosphere (≈30 atm). The system is calibrated with a standard compound of certified calorific value (benzoic acid). The energy content (calories or Jules per gram) is then calculated considering the temperature increase and the sample weight.

The amino acid concentration of preys was determined after hydrolysis in 6 N HCl plus 1% phenol in sealed, evacuated tubes at 110 °C for 24 h by high-performance liquid chromatography (HPLC), according to the Waters Pico Tag method^66^ with pre-column derivatization with phenylisothiocianate using a Waters 2695 separation module (Waters Cromatografía, SA, Spain). The cysteine and methionine contents were determined as cysteic acid and methionine sulphone, respectively, after oxidation with performic acid before protein hydrolysis^67^. Tryptophan was not determined. A Millenium 32 chromatography manager system was used for gradient control and data processing.

### Prey preference and energetic profitability

Prey preference was calculated relating the occurrence of different prey species in the diet to their abundance in the area. Thus, prey preference was calculated for a total of 15 prey species groups whose abundance was known in the study area (Supplementary Appendix S1). Prey preference for coleopteran species was estimated using abundances found for large dung beetles (Supplementary Appendix S1). Total gross energy content of the prey species was calculated by multiplying prey weight (g) by prey energy content (kJ/g). Prey energetic profitability was then calculated as the total gross energy of the prey divided by PPT.

### Statistical procedures

#### Prey provisioning time

Since the activity of both kestrels and prey species vary over the course of a day, different time fractions show different prey provisioning activity (Supplementary Fig. S1). For this reason, we standardised PPT with respect to time of day. Prey provisioning in a given nest shares environment and provisioner but environmental conditions differ across different years. To avoid pseudoreplication in the data, mean PPT was obtained as the mean values in each prey group (marginal means) from a Linear Mixed Model (LMM) in which standardised PPT was the dependent variable, prey species group was included as a fixed factor and nest and year were random factors.

#### Prey provisioning time analyses

All data were analysed using linear models (LMs) in SAS 9.4 software (2002–2012 by SAS Institute Inc., Cary, NC, USA). Residuals from all LMs were checked for normality (Kolmogorov-Smirnov, *P* > 0.2 in all cases). In a first step, we explored the foraging strategy of kestrels when provisioning the nest with food by analysing how prey weight and the difficulty of capturing the prey (independent variables) describe PPT (dependent variable). Secondly, we analysed whether the pattern found in PPT (dependent variable) was correlated with kestrel prey preference (independent variable).

#### Preference and nutrients of the prey

Finally, we analysed the relationship between prey preference as a dependent variable and ecological and nutritional characteristics of the prey groups. For this analysis, we used the sample size for which we obtained nutritional data (n = 11 prey groups). AA content was analysed following a functional criterion by grouping AA as EAA (arginine, histidine, isoleucine, leucine, lysine, methionine, phenylalanine, threonine and valine), SAA (cysteine and methionine) and BCAA (leucine, isoleucine and valine). In addition, AA diversity in each prey group was calculated using the Shannon-Wiener index. In total, we obtained 12 potential explanatory variables: two ecological (prey size and capturability) and 10 nutritional (crude protein, fat, EAA, SAA, BCAA contents, protein-fat ratio, AA diversity, gross energy, total gross energy and energetic profitability). Some of the explanatory variables or predictors are intercorrelated and may even be redundant. For example, BCAA is contained within EAA. To avoid multicollinearity in the model, we analysed these data using partial least squares regressions (PLSR). This is a good technique to identify relevant variables and their magnitude of influence for ecological studies^68^ and is recommended as a substitute for multiple regression, especially when the number of predictor variables is large and there is multicollinearity, as in our case^69^. PLSR strives to identify a few linear combinations (factors) of the original predictor values that describe most of the inherent information in the dependent variable. PLSRs were made in TANAGRA 1.4.50 software^70^. The number of factors were selected by cross-validation using *Q*^2^ (the fraction of the total variation of the dependent variables that can be predicted by a factor). Factor loadings (analogous to Person’s r; the squared loading factor is the percent of variance in the predictor variable explained by the factor), weights (how much the variable predictors contribute to explaining the response variable) and the significant regression coefficients between the response variable and predictors by bootstrapping using 10,000 replications are given.

## Results

### Prey provisioning time, prey size and capturability

Results of the LM showed that larger/heavier prey species and those with higher prey capturability index values took significantly longer to deliver to the nest (Model: *r* = 0.88, *F*_2,12_ = 21.41 *P* < 0.001, *R*^2^ = 0.78,, *n* = 15; Prey weight: *estimate* = 0.254, *S.E*. = 0.09, *F* = 8.28, *95% C. I*. = 0.06–0.44, *P* = 0.014; Prey capturability: *estimate* = 1.796, *S.E*. = 0.76; *F* = 5.50, *95% C. I*. = 0.13–3.46, *P* = 0.037). Prey size and prey capturability were intercorrelated, with larger prey being more difficult to capture (LM, *r*_*s*_ = 0.86, *P* < 0.001), but the variance inflation factor (VIF) for the model was adequate (1.9).

**Figure 1:**
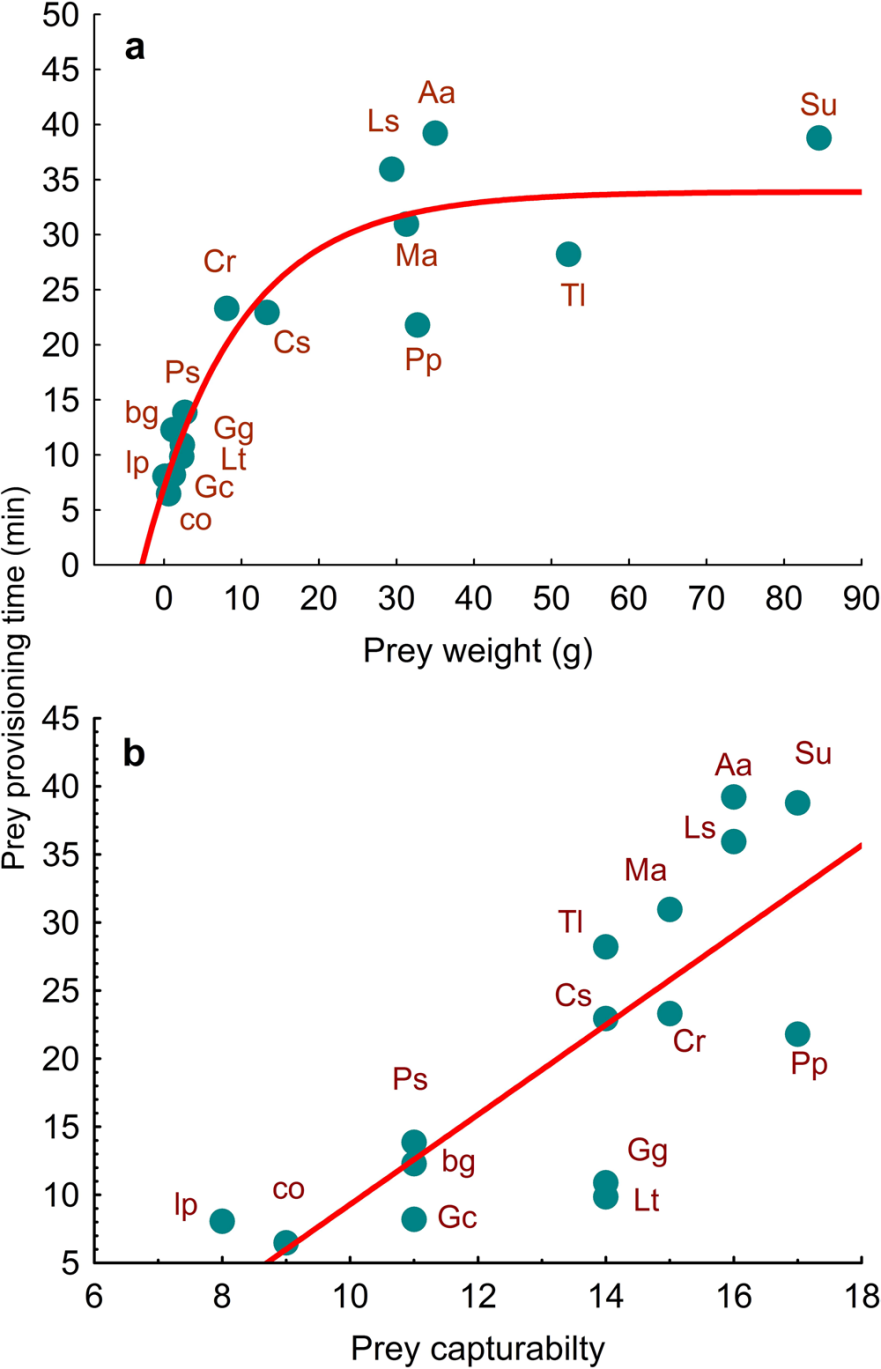
(**a**) Decelerating exponential function describing the relationship between the mean time common kestrels take to provision each prey species and prey weight (PPT = 33.39–26.54/2^(weight/7.74)). (**b**) Linear relationship between prey provisioning time and prey capturability. Aa = *Alauda arvensis*, bg = bush crickets/grasshoppers, co = coleoptera, Cr = *Crocidura russula*, Cs = *Chalcides striatus*, Gc = *Gryllus campestris*, Gg = *Gryllotalpa gryllotalpa*, lp = larvae of Lepidoptera, Ls = *Lacerta schreiberi*, Lt = *Lycosa tarantula*, Ma = *Microtus arvalis*, Pd = *Podarcis hispanica*, Ps = *Psammodromus hispanicus*, Pp = *Pelophylax perezi*, Tl = *Timon lepidus*, Su = *Sturnus unicolor*.

Analysing the relationship between PPT and both potential explanatory variables separately, the relationship between PPT and prey weight was better fitted to a decelerating exponential function (*r* = 0.86, *aR*^2^ = 0.83, *F* = 24.87, *d.f*. = 12, *P* < 0.001, *n* = 15; Fig. 1a), showing a lower *SE* (4.7) than the linear relationship (6.9). The PPT increase began to decline when prey weight exceeded about 10 g. The function curve also showed that kestrels reached a maximum threshold of about 35 min on average to deliver a prey to the nest. Regarding PPT and prey capturability, the relationship was linear (LM, *r* = 0.79, *aR*^2^ = 0.59, *F*_1,14_ = 10.6, *P* < 0.001, *n* = 16; *r* = 0.79, *aR*^2^ = 0.60, *F* = 22.14, *d.f*. = 13, *P* < 0.001, *n* = 15; Fig. 1b).

### Prey preference

**Figure 2:**
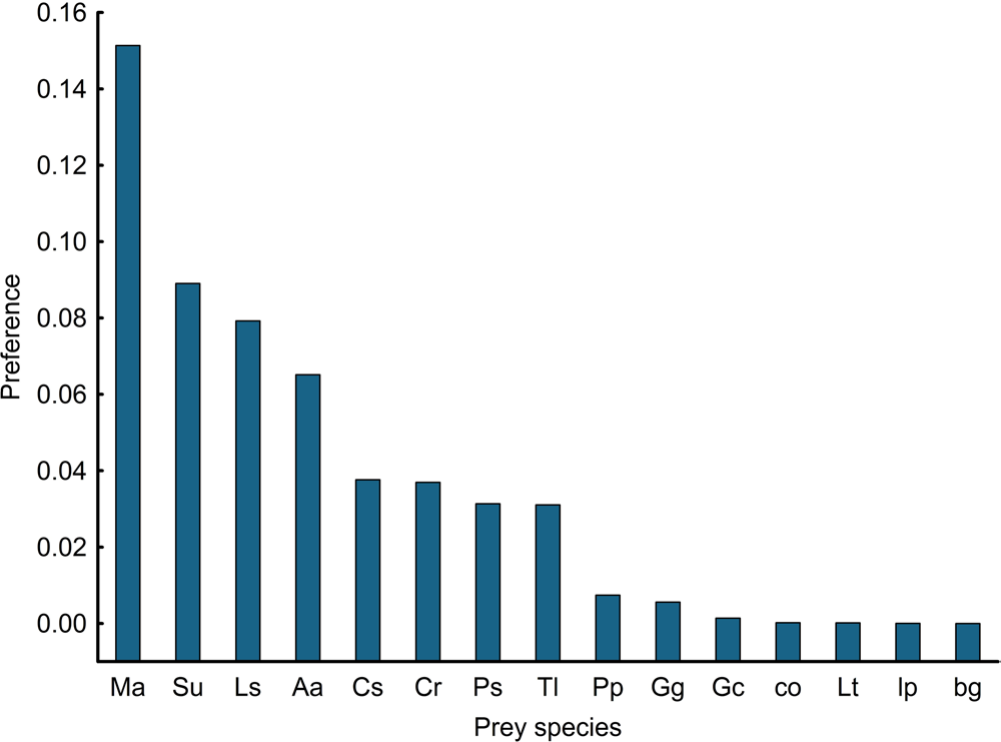
Prey preference of common kestrels. Ma (common vole, *Microtus arvalis*), Su (spotless starling, *Sturnus unicolor*), Ls (Schreiberi’s lizard, *Lacerta schreiberi*), Aa (Eurasian skylark, *Alauda arvensis*), Cs (three-toed skink, *Chalcides striatus*), Cr (common shrew, *Crocidura russula*), Ps (psammodromus lizard, *Psammodromus hispanicus*), Tl (ocellated lizard, *Timon lepidus*), Pp (Perez’s frog, *Pelophylax perezi*), Gg (mole cricket, *Gryllotalpa gryllotalpa*), Gc, (field cricket, *Gryllus campestris*), co (coleoptera), Lt (Mediterranean tarantula, *Lycosa tarantula*), Lp (larvae of Lepidoptera) and bg (bush crickets/grasshoppers).

In decreasing order, common voles, spotless starlings, Eurasian skylarks, greater white-toothed shrews and ocellated lizards were the five most preferred prey species (Fig. 2). Preference for common voles was markedly higher than for the rest of the prey species. In fact, it was close to being an outlier (*Grubbs’s test* = 2.63, *P* = 0.062).

### Prey provisioning time and preference

**Figure 3:**
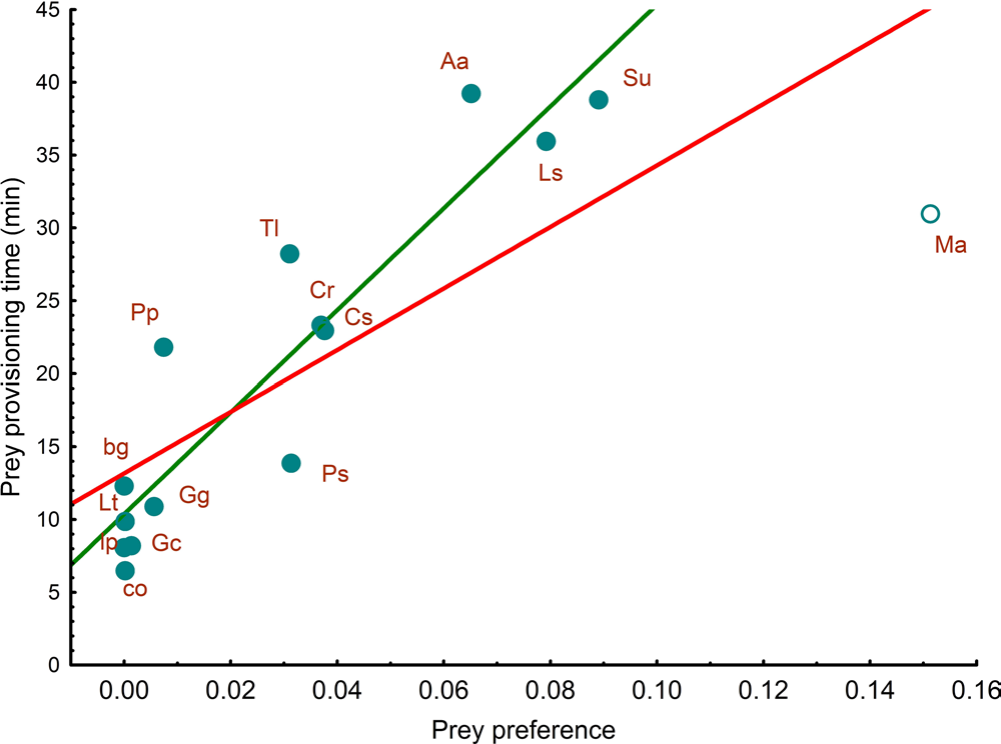
Relationship between prey provisioning time (PPT) and kestrel prey preference. Red and green lines represent the linear function of the regression when common voles (open dot) were included and excluded, respectively. Aa = *Alauda arvensis*, bg = bush crickets/grasshoppers, Cr = *Crocidura russula*, Cs = *Chalcides striatus*, Gc = *Gryllus campestris*, Gg = *Gryllotalpa gryllotalpa*, lp = larvae of Lepidoptera, Ls = *Lacerta schreiberi*, Lt = *Lycosa tarantula*, Ma = *Microtus arvalis*, Pd = *Podarcis hispanica*, Ps = *Psammodromus hispanicus*, Pp = *Pelophylax perezi*, Tl = *Timon lepidus*, Su = *Sturnus unicolor*.

Including common voles, PPT was positively correlated with prey preference (LM, *r* = 0.79, *R*^2^ = 0.62, *F*_1,13_ = 21.6, *P* < 0.001; Fig. 3). Excluding common voles, the models also showed a significant positive correlation between PPT and prey preference (LM, *r* = 0.92 *R*^2^ = 0.85 *F*_1,12_ = 69.9, *P* < 0.001; Fig. 3).

### Nutritional components

The proportion of protein in prey species consumed by kestrels was on average 19.3 ± 1.8 g / 100 g fresh weight (range = 16.2–22.1). The lowest values of protein content were found in field crickets and Perez’s frogs while the highest were found for greater white-toothed shrews and birds (Table 2). Mean fat content of kestrel prey species was 2.4 ± 1.2 g / 100 g fresh weight (range = 0.8–4.6). The lowest fat content was found in Perez’s frogs and the highest values were observed in three-toed skinks, birds and shrews (Table 2). In the case of gross energy content, kestrel prey species have a mean value of 5.7 ± 0.8 Kj / g fresh weight (range 4.3–7.0). Species contributing less energy were the Perez’s frog while birds and mole crickets were the most caloric (Table 2). Considering the biomass of prey consumed at the population level (weighted arithmetic mean), kestrels consumed food with an average proportion of 19.1 g of protein and 2.3 g of fat, resulting in a protein-fat ratio of 8.3. AA profiles for each prey species are shown in the Supplementary Table S2. Aspartic and glutamic acids were the most abundant followed by arginine, and cysteine and methionine were the least abundant. Alanine and tyrosine were particularly abundant in Orthopteran species.

### Preference and prey characteristics

**Figure 4:**
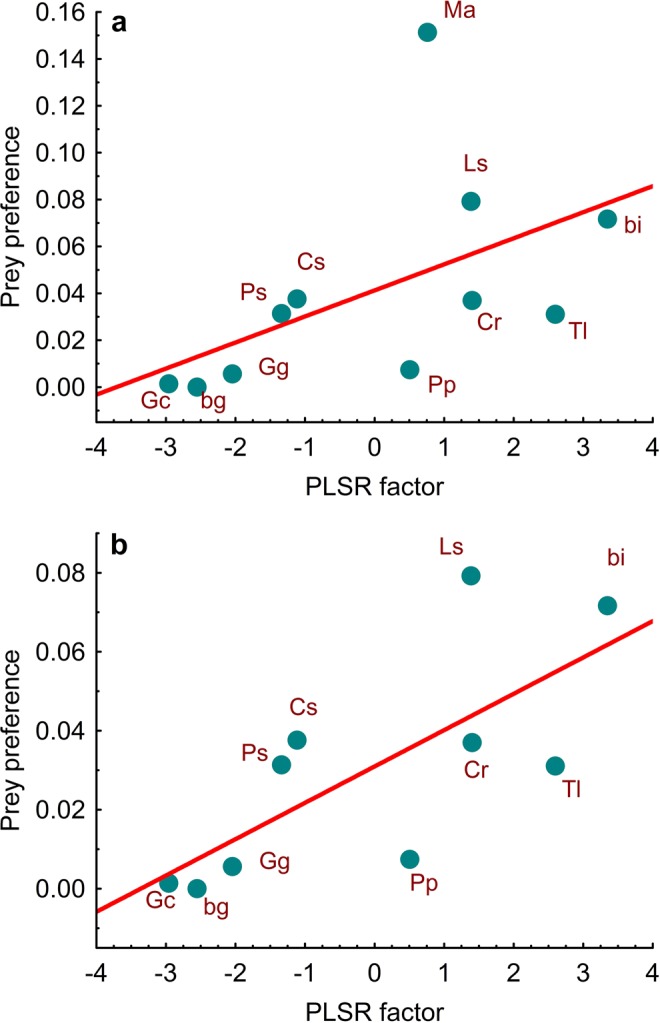
Relationship between prey preference of common kestrels and the factor resulting from PLSR models when voles were included (**a**) and excluded (**b**). bi = birds (*Alauda arvensis* + *Sturnus unicolor*), bg = bush crickets/grasshoppers, Cr = *Crocidura russula*, Cs = *Chalcides striatus*, Gc = *Gryllus campestris*, Gg = *Gryllotalpa gryllotalpa*, Ls = *Lacerta schreiberi*, Ma = *Microtus arvalis*, Ps = *Psammodromus hispanicus*, Pp = *Pelophylax perezi*, Tl = *Timon lepidus*.

Including common voles, the PLSR model resulted in one single factor that explained 38.8% of the variance of all predictor variables and 27.3% of kestrel prey preference. The predictor variables that contributed significantly to explaining prey preference were prey weight, SAA and total gross energy (Table 3). Excluding common voles, the PLSR model also resulted in one single factor that similarly explained 39.8% of the variance of all predictor variables, but in this case the factor explained 58.4% of kestrel prey preference. The predictor variables that contributed significantly to explaining prey preference were prey weight, protein contents, EAA, SAA, AA diversity and total gross energy (Table 3). The relationship between prey preference and factor scores was nearly significant (LM, *r* = 0.52, *R*^2^ = 0.27, *F*_1,9_ = 3.4, *P* = 0.099) when voles were included and significant (LM, *r* = 0.73, *R*^2^ = 0.53, *F*_1,8_ = 9.1, *P* = 0.017) when they were excluded (Fig. 4).

**Table 3:** Results of the PLSR models testing for the effect of prey features on the prey preference of common kestrels. Predictor variables marked in bold and underlined show significant standardised regression coefficients (10,000 replications) for the vole-included and vole-excluded PLSR models, respectively. (*) denotes significant regression coefficients (*P* < 0.05).

Predictor variables	Including voles			Excluding voles		
	Factor loadings	Weights	Regression coefficients	Factor loadings	Weights	Regression coefficients
**Prey weight**	0.42	0.42	0.10*	0.37	0.34	0.12*
Capturability	0.35	0.38	0.09	0.32	0.31	0.11
Protein	0.27	0.20	0.05	0.35	0.38	0.13*
Fat	−0.02	−0.03	−0.01	0.13	0.14	0.05
Protein:fat	0.06	0.01	0.00	−0.04	−0.03	−0.01
Gross energy	0.02	−0.08	−0.02	0.13	0.12	0.04
Energ. profitability	0.40	0.33	0.08	0.36	0.29	0.10
EAA	0.38	0.39	0.09	0.39	0.43	0.15*
**SAA**	0.36	0.40	0.10*	0.36	0.38	0.14*
BCAA	−0.07	−0.01	−0.03	−0.03	−0.10	−0.04
AA diversity	0.22	0.18	0.04	0.25	0.21	0.07*
**Total gross energy**	0.42	0.42	0.10*	0.39	0.38	0.13*

## Discussion

The time devoted by kestrels to provisioning the nest with a given prey was related to size (weight) and capturability of the prey species, with both variables explaining a high percentage of the variance in prey provisioning time. Smaller prey took less time to be provisioned than larger prey. However, the relationship between provisioning time and prey weight was not linear, but was adjusted to an exponential decay (half-life increasing form) linear function. Prey weight showed high explanatory power (steep slope) for species up to 13 g (arthropods and small lizards), while prey above 30 g had a low capacity to explain the time devoted to provisioning the nest with a prey. This result clearly shows that larger prey species are more profitable in terms of biomass provided per unit of time. It suggests that for the prey sizes that kestrels can capture and take to the nest, times longer than a certain threshold of about 35 min render them unprofitable or kestrels seldom take more than 35 min to capture a large prey. A time threshold may be expected within a central place framework, since food provisioning is predicted to be constrained by the time needed for self-feeding and, likewise, the distance that kestrels need to travel to hunt can affect the size and provisioning time of the prey^71^. In kestrels, it has been reported that breeding individuals maximise daily energy gain during the chick-rearing phase by adjusting hunting time below a predicted physiological maximum to guarantee energetic balance^72,73^. Accordingly, our results suggest that optimal provisioning eludes foraging events longer than the time-energy budget of individuals.

Our results also indicate that the load-size effect does not explain provisioning time in the case of large prey. However, prey provisioning time varied proportionally with prey capturability and larger prey were more difficult to capture (starlings, skylarks, Perez’s frogs or Schreiber’s lizards). It is worth noting that our prey capturability index included parameters such as prey abundance and ubiquity that affect the probability of encounters. Large prey species in our study area tend to be less abundant and some of them, such as common voles, spotless starlings, Perez’s frogs and Schreiber’s lizards, were spatially localised in specific areas. Furthermore, other characteristics, such as speed flight, are expected to be higher, for larger species. All of these ecological characteristics make it harder for large prey to be captured.

S. Moore^13^ proposed that low selectivity for prey also increases feeding rate, therefore shortening the between-feeding interval, while a more restrictive selection to find and capture preferred prey increases the time devoted to provisioning the nest with food. Our results seem to support this idea, since prey provisioning time was positively correlated with kestrel prey preference. However, the positive correlation found between prey provisioning time and prey capturability suggests that the difficulty of capturing a prey also impacts the time that kestrels spend in provisioning the nest with a given prey species. More preferred prey types are also less abundant, farther and less ubiquitous (i.e. higher difficulty of capture), for which less preferred prey offer a higher probability of encounters, therefore, diminishing between feeding intervals. Unlike size, prey provisioning time varied with prey capturability among large prey (linear relationship) as well, indicating that capturability of the prey better predicts the time the kestrels devote to provisioning the nest with food compared to prey size.

Our results demonstrate the explanatory capacity that an index of prey capturability has for the time devoted to provisioning the nest with a given prey species. Many morphological, behavioural and ecological characteristics of species have evolved under the pressure of predation, affecting prey vulnerability and the foraging strategies of predators^74^. Antipredatory prey traits influence both energy expenditure and time spent by predators in every step of the predation sequence (prey encounter, detection, pursuit, capture, handling and consumption), thus influencing foraging decisions at the individual level^75–77^. Despite its relevance in how animals optimise food acquisition, little effort has been made to measure the potential of capturability of prey species in order to integrate this variable into optimal foraging models, particularly under the central place scheme. Capturability indexes, like the one we use here, are generally based on a human perception of the prey characteristics that impede predation. This lends an inevitable degree of subjectivity to the indexes. For that reason, we included eight variables under the premise that the more features included, the more objective the index. The index we use in this study is an easy index to measure, but requires some field experience in the study area.

Kestrel prey preference at the chick-rearing stage was clearly associated with weight and nutritional content of the prey. When voles are considered in the analyses, the average prey preferred by kestrels is large, caloric and has high contents of sulphur AA. When voles are excluded from the models, kestrel preference is also explained by protein, essential AA and AA diversity. The strong preference for voles (numerically distant from the rest of the data) and the high weight of this prey species support a PLSR factor in which prey weight and total gross energy (probably also as a result of prey weight) play an important role as concluded by the factor loadings of both variables. A relevant point of the results is that the vole-included PLSR factor explains 31.1% less variance in kestrel prey preference than the vole-excluded PLSR factor, which indicates that kestrel prey preference is notably better defined by this latter factor. In granivorous, frugivorous and insectivorous bird species it has been shown that individuals choose food based on nutritional components^78–80^. Furthermore, recent studies in carnivorous-piscivorous birds show that the nutritional variability found in the food consumed potentially presents an opportunity to select an optimal diet, with respect to the protein-fat ratio^81^. Our results indicate that the nutritional composition of the prey is closely correlated to prey preference suggesting that carnivorous-insectivorous bird species may be able to choose food according to its nutritional value and specific nutrients, rather than a particular protein-fat ratio, as deduced from the negligible influence of the protein-fat ratio in prey preference of kestrels. The possibility that predators may search for prey that contains complementary nutrients in order to balance the diet has also been suggested, e.g.^81^. With respect to this, the consumed prey show wide ranges of variation in nutrients and protein-fat ratios, so kestrels could prey in an environment where compensatory nutrients are obtainable.

The common kestrel has traditionally been considered a vole specialist^60^, since voles are the main prey consumed in studied populations from northern and central latitudes of Europe^61,72,82^. Studies in southern latitudes differ from this view indicating a wide range of prey species present in the diet of kestrels (see^56^ and references therein). Compared to the morphology of bird-eating falcons, the common kestrel shows a shorter wing length relative to tail length as well as shorter toes, with both traits corresponding to phenotypes more specialised in capturing reptiles and mammals^61^.

In our population, the common vole (the only vole species in the area with epigean foraging and diurnal habits) is the fourth most consumed prey species representing 8% of occurrence in the diet, but contributing the most biomass (38%; Supplementary Table S1). Additionally, the abundance of common voles is key in the demographic models of kestrels in our study area^63^. Our results show that this species is strikingly preferred by kestrels in our population, indicating that even with a high plasticity in prey consumption^56,62^, common kestrels selectively search for voles in the area. This foraging strategy would be expected if predator-prey coevolution promoted morphological and physiological adaptations in kestrels to capture voles, resulting in the highest efficiency and energy rewards for this species. Our study certainly shows that when voles are included in the model, prey characteristics, such as weight and total gross energy (closely associated with weight), contribute significantly to explaining kestrel preference, with the models without voles showing a greater influence of nutritional variables. However, bird prey species (starlings and skylarks) were the most preferred prey species after voles, which seems apparently contradictory for a rodent specialist predator. Nonetheless, as in other kestrel populations, birds predated upon by kestrels during the breeding season are almost entirely fledglings^82^. Similarly, in our population adult birds are anecdotally found in kestrel nests as prey remains (personal observations). The naïve antipredatory behaviour of the fledglings makes them a fruitful food resource at the middle-end of the breeding season, during which specific morphological adaptations are not necessarily required. This may explain the high values observed in kestrel preference for birds, considering this result is derived from the opportunistic exploitation of a seasonally flourishing and relatively cheap food. After fledglings, reptiles and small mammals showed the highest values of preference, in agreement with that expected based on kestrel phenotypes. However, our results do not explain the high vole preference of kestrels from a nutritional viewpoint. Several explanations may be behind this result. The observed behaviour may be the result of a prevalent foraging strategy derived from morphological adaptations in predominantly vole predatory species, even when a large variety of alternative and apparently more nutritious prey is available. The lower proportion of proteins and fat found in voles compared to other prey species may be compensated for with the high biomass supplied. A different explanation would be that kestrels may obtain important nutritional components (other than fat, protein and amino acid contents) from voles that were not analysed in this study. Trophic niche breadth is a cornerstone in ecological theory and knowing the position that individuals, populations and species occupy in the specialist-generalist diet axis is considered essential to understand eco-evolutionary processes from individual fitness and population dynamics to speciation patterns or species distribution^2,20^. In this sense, our study highlights the importance of the nutritional approach for a better understanding of the generalism and specialism concepts as recently advanced^83^.

In summary, from the perspective of the central place theory, our study reveals that for a predator species, prey load-size has a low explanatory power for large prey and that prey capturability plays an essential role in describing foraging strategies. This highlights the importance of considering capturability indexes in optimal foraging models. In addition, our study shows that larger prey (over 30 g in the case of kestrels) are more profitable in terms of the biomass provided to offspring than smaller prey species and that preferred prey take longer to be captured and provisioned to the nest. Furthermore, we can conclude that predator foraging may use strategies aimed at acquiring required nutritious food by choosing prey species based on their nutritional and energetic profiles. Analysing the nutritional composition of food is therefore essential to understand foraging ecology and the optimal foraging strategies of a species, including predators.

## Supplementary information

Supplementary information.
