# Supplementary material for: Foraging strategy of a carnivorous-insectivorous raptor species based on prey size, capturability and nutritional components
Source: Sci Rep. 2020 May 5;10:7583. doi: 10.1038/s41598-020-64504-4 (PMC7200729; doi:10.1038/s41598-020-64504-4)
Supplement: Supplementary file 1 — Supplementary information. [file 41598_2020_64504_MOESM1_ESM.pdf]

Supplementary material

Foraging strategy of a carnivorous-insectivorous raptor species based on prey size, capturability and nutritional components

Juan A. Fargallo[1*], Juan Navarro-López[1], Patricia Palma-Granados[2], and Rosa M. Nieto[2]

[1]*Departamento de Ecología Evolutiva, Museo Nacional de Ciencias Naturales-CSIC, José Gutiérrez Abascal 2, 28006 Madrid, España.*

[2]*Department of Physiology and Biochemistry of Animal Nutrition, Estación Experimental del Zaidín-CSIC, Camino del Jueves s/n, 18100 Armilla, Granada, España.*

* fargallo@mncn.csic.es

Appendix S1

Prey abundance

Abundances were estimated as an approximate number of individuals in the whole study area (3,000 ha) using the following methodologies:

Abundances of field crickets *Gryllus campestris*, mole crickets *Gryllotalpa gryllotalpa* and wolf spiders *Lycosa tarantula* were estimated using direct counts of active borrows in 1,654 pastureland squares of 5 x 5 m between 2002 and 2015 (116 ± 20.4, range 78 – 176 squares each year). In the same squares, the number of Spanish Psammodromus *Psammodromus hispanicus* individuals was also recorded. Results showed densities of 1.5 ± 2.5 individuals / 25 m^2 (range = 0 – 23) for field crickets, 0.6 ± 1.0 individuals / 25 m^2 (range = 0 – 9) for mole crickets, 0.2 ± 0.7 individuals / 25 m^2 (range = 0 – 6) for wolf spiders and 0.05 ± 0.2 individuals / 25 m^2 (range = 0 – 2) for Spanish Psammodromus. These species were present in all habitats found in the study area (Navarro-López and Fargallo 2015).

The abundance of all coleoptera species consumed by kestrels could not be estimated in the field. However, it was possible to estimate the abundance of large (> 15 mm) dung beetles species, which are the most consumed by kestrels. Nine large dung beetle species (*Bubas bubalus*, *Cheironitis hungaricus*, *Copris lunaris*, *Gymnopleurus flagellatus*, *Onitis belial*, *Scarabaeus laticollis*, *Anoplotrupes stercorosus*, *Geotrupes stercorarius* and *Trypocopris pyrenaeus*) have been found in our study area (Martín-Piera et al. 1992) of which 161

individuals per 1,000 g of fresh dung were estimated (data kindly provided by Jorge M. Lobo and extracted from Martín-Piera et al. 1992). Considering that one cow excretes on average 24 kg of dung per day (Haynes and Williams 1993) and that in our area there is a density of 318 cows per 1000 ha (pers. obs.), the amount of large dung beetles were estimated in 2,940 ha, after excluding about 60 ha of ungrazed fenced pastures free of cattle.

The abundance of lepidopteran larvae (caterpillars) was estimated during 2016 following the same method as above in 119 pastureland squares of 5 x 5 m randomly distributed in the study area. Larva density was 3.9 ± 11.2 individuals / 25 m^2 (range = 0 – 70). Lepidopteran larvae are also present in all habitats of the study area.

The abundance of *Acrididae* and *Tettigoniidae* species was estimated by direct counts of bush crickets and grasshoppers captured using a 0.28 m diameter sweep net. Animals were captured by two parallel sweeps (one in the opposite direction of the other) along two arcs of about 5 m each, for which the swept area was about 2.8 m^2. Each sweeping point was 10 strides apart. In total, 2309 points were swept in different pastureland habitats from 2002 to 2016 (mean = 154 ± 3.4 each year; range = 150 - 158). Results showed an average of 4.3 ± 6.8 individuals / 2.8 m^2, range = 0 – 72. *Acrididae* and *Tettigoniidae* species are present in all habitats of the study area.

For common voles *Microtus arvalis*, greater white-toothed shrews *Crocidura russula* and ocellated lizards *Timon lepidus* abundance estimates were based on individual numbers recorded by trapping in the study area (see Fargallo et al. 2009) and following the capture-mark-recapture Schnabel index (Krebs 1999). Mean densities resulted in 101 voles/ha, 33.8 shrews/ha and 13.3 lizards/ha. Voles and shrews are rarely observed outside of ungrazed pastures, with this habitat representing 2% (60 ha) of all habitats present in the study area (Navarro-López and Fargallo 2015). Lizards were only observed in ungrazed pasture, rocky land, broom scrubland and oat pasture representing 30% (900 ha) of all habitats. Calculations of abundance for these three species were made considering the habitats where they were present.

The abundance of Schreiber's green lizards, *Lacerta schreiberi*, was estimated using transects made in the broom scrubland during five years (from 2010 to 2012, 2014 and 2015). This is the only habitat where lizards were observed that represents 5% (150 ha) of the study area. A line transect 1.3 km in length were walked every year in mid-June in sunny days and starting at 8:00 am solar time. The observer (J.A.F.) walked slowly about 1km/h counting all lizards detected within a 4 m band. Transect could be repeated a couple of weeks later in years 2010 to 2012. Mean density resulted in 8.5 individuals/ha.

The abundance of three-toed skinks *Chalcides striatus* was calculated based on the transects made in the study area from 2008 to 2012 and the years 2014 and 2015. Every year, two transects 300 m in length were walked in two different ungrazed pasture plots during three days in mid-June. Transects were walked once in the morning (8:00 – 10:00 am solar time) and once in the evening (5:00 – 7:00 pm solar time) during three days. In addition, one more transect 1.2 km in length was walked every year in early July in a plot including ungrazed, evergreen and oat pastures starting at 8:00 am solar time. The observer (J.A.F.) walked slowly about 1km/h counting all skinks detected within a 3 m band. Mean density resulted in 9.5 skinks/ha. Lizards were only observed in ungrazed, evergreen, oat pastures and in broom scrubland, representing 35% (1050 ha) of all habitats. Calculations of abundance for these three species were made considering the habitats where they were present.

To calculate the abundance of Perez's frogs *Pelophylax perezi*, counts carried out in the study area in 1994 were used. The number of individuals was estimated through indirect calculations of adult frogs observed in river ponds. These ponds are the only natural bodies of water present at the end of summer. Frogs were counted in nine randomly selected river ponds varying from 5 to 43 m long (mean = 16.7 ± 11.8 m) and estimated three times in August, September and October. Counts were made using binoculars and from a distance that did not provoke escape responses (Martín et al. 2006). A mean of 23.2 ± 16.8 (n = 9 x 3, range 0 - 70) frogs were counted in each pond (J.A. Fargallo, and E. Soto-Largo, unpublished data). Final calculations were made by multiplying the mean number of frogs by 145 ponds present in 28.3 km of the Voltoya River in the study area.

Abundances of spotless starlings *Sturnus unicolor* and Eurasian skylarks *Alauda arvensis* were estimated by means of observation points along an 11-km road crossing the valley in a similar way as described by Martínez-Padilla and Fargallo (2008). Abundances found at these points were extrapolated to the rest of the study area where both species are present.

<u>Category 1: very abundant prey species (more than 200,000 individuals in the study area)</u>

Calculations resulted in ≈46 million individuals of **Acrididae-Tettigoniidae** (1.54 individuals / m^2 on average), ≈1.8 million **field crickets** (0.060 individuals / m^2 on average), ≈720,000 **mole crickets** (0.024 individuals / m^2 on average), ≈252,000 **wolf spiders** (0.0084 individuals / m^2 on average), ≈4.7 million **caterpillars** (0.156 individuals / m^2 on average) and ≈3.7 million dung beetles (0.122 individuals / m^2 on average).

Category 2: abundant prey species (between 200,000 and 10,000 individuals in the study area)

Calculations resulted in ≈60,000 **psammodromus lizard** and ≈9,993 **three-toed skinks** in the study area.

Category 3: low abundance prey species (less than 10,000 individuals in the study area)

Calculations resulted in ≈11,970 **ocellated lizards**, ≈6,060 **common voles**, ≈1,275 **Schreiber's green lizards**, ≈3,364 **Perez's frogs,** ≈2,028 **greater white-toothed shrews**, ≈660 **Eurasian skylarks** and ≈247 **spotless starlings**.

References

Fargallo, J. A., Martínez-Padilla, J., Viñuela, J., Blanco, G., Torre, I., Vergara, P., & De Neve, L. (2009). Kestrel-prey dynamic in a Mediterranean region: the effect of generalist predation and climatic factors. *PLoS One*, *4*(2), e4311.

Haynes, R.J. & Williams, P.H. (1993) Nutrient cycling and soil fertility in the grazed pasture ecosystem. *Advances in Agronomy*, **49**, 119–199.

Martín, J., Luque-Larena, J. J., & López, P. (2005). Factors affecting escape behavior of Iberian green frogs (Rana perezi). *Canadian Journal of Zoology*, *83*(9), 1189-1194.

Martínez-Padilla, J., & Fargallo, J. A. (2008). Fear in grasslands: the effect of Eurasian kestrels on skylark abundances. *Naturwissenschaften*, *95*(5), 391-398.

Krebs, C. J. (1989). *Ecological methodology* (No. QH541. 15. S72. K74 1999.). New York: Harper & Row.

Martín-Piera, F., Veiga, C. M., & Lobo, J. M. (1992). Ecology and biogeography of dung-beetle communities (Coleoptera, Scarabaeoidea) in an Iberian mountain range. *Journal of Biogeography*, 677-691.

Navarro-López, J., & Fargallo, J. A. (2015). Trophic niche in a raptor species: the relationship between diet diversity, habitat diversity and territory quality. *PloS one*, *10*(6), e0128855.

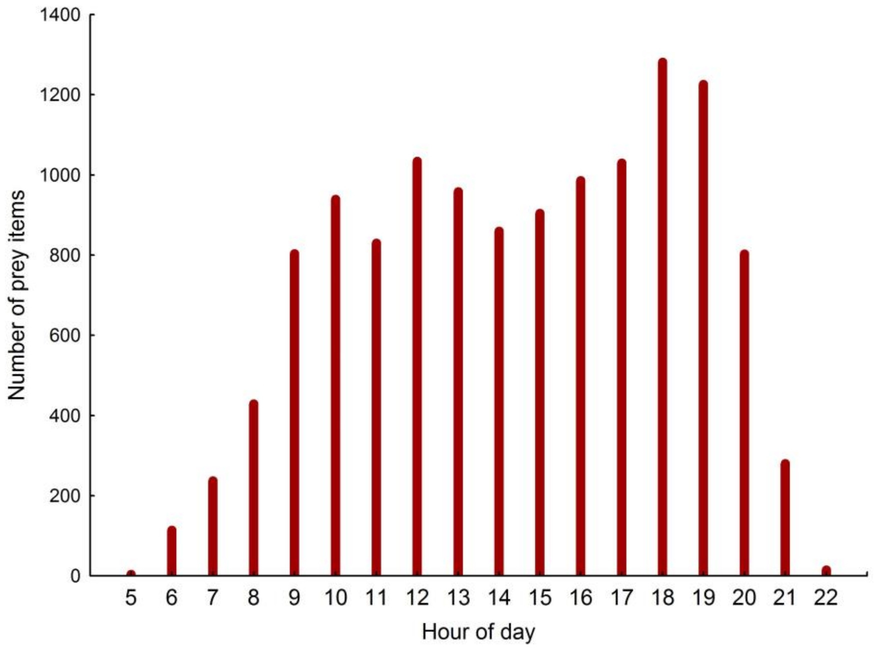

Fig. S1. Number of prey items brought to the nest at different times of the day.

Table S1. Total numbers, percentage and biomass of prey items delivered by common kestrel *Falco tinnunculus* parents to the nest over a nine-year study period (2006-2014). The minimum identified taxon level is shown.

Prey species	N (%)	% biomass
**Mammals**	1126 (8.8)	38.6
Apodemus sylvatica	8 (0.1)	0.2
Crocidura russula	82 (0.6)	0.8
Microtus arvalis	1034 (8.1)	37.48
Talpa occidentalis	1 (0.0)	0.12
Mus musculus	1 (0.0)	0
**Birds**	146 (1.1)	7.6
Alauda arvensis	43 (0.3)	1.61
Anthus campestris	2 (0.0)	0.1
Emberiza calandra	1 (0.0)	0.06
Falco tinnunculus	1 (0.0)	0.14
Lanius senator	1 (0.0)	0.1
Linaria cannabina	4 (0.0)	0.1
Motacilla flava	5 (0.0)	0.1
Passer domesticus	5 (0.0)	0.16
Petronia petronia	5 (0.0)	0.1
Saxicola rubicola	1 (0.0)	0
Sturnus unicolor	22 (0.2)	2.12
Sylvia atricapilla	1 (0.0)	0
Passerines (unidentified)	10 (0.1)	0.6
Birds (unidentified)	45 (0.4)	2.46
**Reptiles**	2965 (23.2)	34.4
Chalcides striatus	397 (3.1)	6.3
Timon lepidus	399 (3.1)	18.28
Lacerta schreiberi	109 (0.9)	3.76
Podarcis hispanica	101 (0.8)	0.24
Psammodromus hispanicus	1948 (15.2)	5.53
Large Lizard a	6 (0.0)	0.33
Small lizard b	5 (0.0)	0.01
**Amphibians**	44 (0.3)	1.6
Triturus marmoratus	3 (0.0)	0.03
Pelophylax perezi	34 (0.3)	1.29
Pelobates cultripes	7 (0.1)	0.27
**Arthropods**	8469 (66.3)	17.5
Lycosa tarantula	47 (0.4)	0.13
Grillus campestris	2511 (19.6)	3.67
Acrididae	278 (2.2)	0.2
Tettigoniidae	450 (3.5)	0.81
G. gryllotalpa	4168 (32.6)	11.84
Orthoptera (unidentified)	5 (0.0)	0.01
Neuroptera	1 (0.0)	0
Mantodea	4 (0.0)	0.01
Lepidoptera	1 (0.0)	0
Coleoptera	760 (6.0)	0.6
Insecta (unidentified)	110 (0.9)	0.23
Insecta (larvae)	134 (1.0)	0.02
Unidentified prey item	29 (0.2)	0.24
**Total prey items**	**12779**	

Table S2. Amino acid content (mg AA / 100 g total AA) in each prey species or prey group. bcg = bush crickets-grasshoppers, bi = birds (*Alauda arvensis* + *Sturnus unicolor*), Cr = *Crocidura russula*, Cs = *Chalcides striatus*, Gc = *Gryllus campestris*, Gg = *Gryllotalpa gryllotalpa*, Ls = *Lacerta schreiberi*, Ma = *Microtus arvalis*, Psh = *Psammodromus hispanicus*, Pp = *Pelophylax perezi*, Tl = *Timon lepidus*.

Species	Ala	Arg	Asp	Cys	Glu	Gly	His	Ile	Leu	Lys	Met	Phe	Pro	Ser	Thr	Tyr	Val
Ma	6.0	8.2	12.3	1.3	17.5	6.3	2.8	3.7	8.0	6.9	1.2	3.7	4.7	4.7	4.0	3.7	4.9
Cr	6.0	9.7	7.9	1.5	13.1	7.4	2.8	3.8	7.7	7.1	1.8	4.3	6.8	5.6	4.8	4.4	5.2
bi	6.2	9.7	10.8	0.8	16.9	6.2	2.7	4.0	8.0	7.2	1.9	3.8	4.8	4.2	4.0	3.7	5.0
Cs	6.5	9.5	10.5	0.9	18.9	7.9	3.2	3.8	7.3	6.5	0.8	4.1	3.9	4.4	3.7	3.5	4.7
Tl	6.1	9.2	10.7	0.6	16.4	6.9	3.3	4.1	7.6	7.3	2.0	3.6	5.3	4.5	3.8	3.8	4.8
Ls	6.0	8.8	10.8	0.7	17.5	7.9	3.1	4.0	7.1	6.9	2.1	3.5	5.2	4.4	3.9	3.6	4.5
Psh	6.0	8.8	11.5	0.6	18.0	6.7	3.0	3.9	7.9	6.7	1.9	3.5	4.3	4.6	3.9	3.7	4.7
Pp	5.9	6.8	13.7	0.4	19.5	6.6	3.2	3.9	7.2	7.3	1.8	3.5	4.4	4.3	3.8	3.3	4.3
Gc	10.4	7.2	13.3	0.6	15.7	5.5	3.2	3.8	7.2	4.9	1.2	2.8	4.7	5.0	3.3	5.3	6.0
Gg	10.5	7.1	13.2	0.5	16.7	5.3	3.6	3.8	7.1	4.6	1.5	2.5	4.5	5.3	3.2	5.3	5.5
bcg	9.2	7.6	11.9	0.5	16.3	5.6	2.9	3.8	7.5	5.4	1.2	2.9	5.0	5.0	3.6	5.7	6.0
